# Enteric Pathogens Risk Factors Associated with Household Drinking Water: A Case Study in Ugu District Kwa-Zulu Natal Province, South Africa

**DOI:** 10.3390/ijerph19084431

**Published:** 2022-04-07

**Authors:** Colette Mmapenya Khabo-Mmekoa, Bettina Genthe, Maggy Ndombo Benteke Momba

**Affiliations:** 1 Department of Biomedical Sciences, Arcadia Campus, Tshwane University of Technology, 175 Nelson Mandela Avenue, Arcadia, Pretoria 0001, South Africa; 2 Department of Environmental, Water and Earth Sciences, Arcadia Campus, Tshwane University of Technology, 175 Nelson Mandela Avenue, Arcadia, Pretoria 0001, South Africa; 3 Water Institute, Department of Microbiology, Stellenbosch University, Private Bag X1, Matieland 7602, South Africa; bettinagenthe@gmail.com

**Keywords:** HIV/AIDS, health risk assessment, drinking water quality

## Abstract

The occurrence of diarrheal infections depends on the level of water and sanitation services available to households of immunocompromised individuals and children of less than five years old. It is therefore of paramount importance for immunocompromised individuals to be supplied with safe drinking water for better health outcomes. The current study aimed at ascertaining the probability of infection that *Escherichia coli*, *Salmonella typhimurium*, *Shigella dysenteriae*, *Vibrio cholerae*, and rotavirus might cause to rural dwellers as compared to urban dwellers. Both culture-based and molecular-based methods were used to confirm the presence of target microorganisms in drinking water samples, while Beta-Poisson and exponential models were used to determine the health risk assessment. Results revealed the presence of all targeted organisms in drinking water. The estimated health risks for single ingestion of water for the test pathogens were as follows: 1.6 × 10^−7^ for *S. typhimurium*, 1.79 × 10^−4^ for *S. dysenteriae*, 1.03 × 10^−3^ for *V. cholerae*, 2.2 × 10^−4^ for *E. coli* O157:H7, and 3.73 × 10^−2^ for rotavirus. The general quantitative risk assessment undertaken in this study suggests that constant monitoring of household container-stored water supplies is vital as it would assist in early detection of microbial pathogens. Moreover, it will also allow the prompt action to be taken for the protection of public health, particularly for immunocompromised individuals and children who are prone to higher risk of infections.

## 1. Introduction

There is an assumption that water complying with the safety regulations is safe for human consumption. However, the reality is more complex. To declare that the water is safe for human consumption, it must be tested for microbial indicators to ensure their absence in the drinking water. A major shortcoming has been linked with the presence of pathogens that resist disinfection, despite the indicator organisms not being present [1]. This translates to the possibility of water complying with the water quality standards yet containing pathogens that are unsafe for human consumption. Infections with bacterial pathogens have been reported to cause a heavy burden on human beings; furthermore, the evidence for the virulence of enteric pathogens is well-documented [2,3,4,5,6,7,8]. Bacterial gastrointestinal infections continue to cause high morbidity and mortality that contribute to the economic loss in some parts of the world [9]. Immunocompromised individuals and very young children are at a greater risk of contracting serious waterborne infections than immunocompetent individuals [10,11,12]. Additionally, aggravating the situation is the loss of normal intestinal flora due to antibiotics prescribed for the treatment of the disease [13].

To understand the risk of exposure to enteric pathogens, the pathogen involved and the product with which it is associated (i.e., food or water) must be identified, as well as the timing and duration of exposure, and the population at risk [12,13]. This will provide strong evidence linking the exposure and incidence of infections in a population. The pathogen concentration required to infect a host (infective dose) varies dramatically across species. In addition, not every exposure to a pathogen in water will result in infection, and not all individuals are equally susceptible [14]. It is accepted that there may be no infections at lower doses; however, at higher doses, the response may occur at a lower probability. It has also been demonstrated that the level of infection increases each time a pathogen causes an infection in the host [15,16,17,18]. 

The health risk assessment processes are defined as a qualitative or quantitative description of the probability of adverse effects that may result from exposure to a microorganism or its toxin [19,20,21,22]. Factors affecting the probability of infection related to the microorganisms include: virulence and infectivity, genetic material and/or dosage, die-off rates, and attenuation and dilution factors. In the human host, these factors include age, pregnancy, nutrition, overall health, medication status, concurrent infections, immune status, and previous exposure history, whilst the population characteristics include population immunity and access to medical care [23,24,25]. 

The prevalence of diarrhoea has been reported in immunocompromised individuals such as children, the elderly, and HIV/AIDS-infected individuals [26]. Outbreaks of pathogenic *Escherichia coli* such as *E. coli* O157: H7 have been associated with the consumption of contaminated municipal water, well water, and recreational waters. These have resulted in the morbidity rates of around 0.51 [27] and the case fatality ratios ranging from 0.005 to 0.0083 [28]. The majority of *Salmonella* infections are also due to the ingestion of contaminated food or water. In people with normal gastrointestinal tracts and immune systems, it requires an estimated 1 million to 1 billion *Salmonella* cells to be ingested in order to cause infection as normal human stomach acid kills large numbers of these bacteria [28,29]. The strains of *Vibrio* that usually produce outbreaks of epidemic cholerae comprises toxin-producing strains of the O1 serovar and the reported serovar, O139 [30]. Serotypes of *V. cholerae* belonging to non-O1/non-O139 are believed to also cause sporadic cases of limited outbreaks of diarrhoea in humans [30]. The severity of this disease depends on several factors, which include personal immunity, inoculum, the gastric barrier, and the blood group [30]. *Shigella* annually was responsible for 1 million death in all ages [31]. 

Rotavirus is considered to be among the most common causes of infant gastroenteritis, resulting in mortality worldwide and triggering up to 197,000–233,000 deaths annually [32]. Rotavirus, transmitted by the faecal–oral route, are shed in high concentrations in the faeces of infected persons, and become infectious for humans at relatively low doses. The period of illness is acute, and the symptoms often start with vomiting, followed by four to eight days of profuse diarrhoea [33]. This study aimed at ascertaining the probability of infection in relation to the infectious dosage that the participants will be exposed to when drinking water contaminated with *Escherichia coli*, *Salmonella typhimurium*, *Shigella dysenteriae*, *Vibrio cholerae*, and Rotavirus.

## 2. Materials and Methods

### 2.1. Ethics Clearance Approval

Prior to conduct the present study, the ethics clearance was obtained not only from the Tshwane University of Technology (TUT) where the study was registered, but also from the Provincial Department of Health in KwaZulu-Natal and the relevant state hospital clinics, where the patients’ health status was confirmed. The selection criteria of the participants included HIV- and non-HIV-infected individuals with diarrhoeal diseases who attended these clinics to get treatments and who also lived in the study areas. The informed consent to participate in the study was obtained from patients, mothers of babies, or patient guardians who granted permission to collect the stool specimens of HIV/AIDS- and non-HIV-infected individuals by the hospital nurses. Only the nurses of the selected state hospitals were able to know the identification and confidentiality of the participants in this study. The household drinking water samples were only collected in the areas where the patients reside, and informed consent was obtained from the owners of houses. Prior to sample collections, a clear justification of the aim and objectives of the study was provided to the study participants. The project expectations and respective obligations by both the participants and investigators were explained, and any questions were answered. The participants were not subjected to risks of any kind as a result of the project.

### 2.2. Study Area and Sample Collection

The study was conducted at Ugu District Municipality, located to the Province of KwaZulu-Natal in South Africa. The area was identified based on the study participant selection of 164 confirmed HIV/AIDS as well as 44 HIV/AIDS-negative individuals, both with diarrhoea. Household drinking water samples were collected from the area sites where the study participants lived. 

The exposure assessment was conducted at a consumer level where consumption of water by confirmed HIV/AIDS patients with diarrhoea and HIV/AIDS-negative patients with diarrhoea were considered for the risk assessment. As there were no data relating the susceptibility to HIV/AIDS-positive individuals to various pathogens, it was assumed that all exposed individuals had an equal chance of infection. 

A total of 1867 drinking water samples were aseptically collected using sterile 2 L sterile plastic bottles, which contained 1 mL of 10% sodium thiosulphate (Na_2_S_2_O_3_) per litre for preservation. The drinking water samples were collected from the end-user point, which consisted of household container-stored water in the rural settlement areas of Boboyi, Bomela, and Gamalakhe and of household tap water in the urban areas of Anneline, Hibberdene, Margate, and Port Shepstone of the Ugu District Municipality, KwaZulu-Natal Province, South Africa. Collected drinking water samples were aseptically transported to the National laboratory Health Centre located within the premises of Portshepstone Hospital in cooler-boxes containing ice and were analysed within 12 h. 

### 2.3. Assessment of the Household Drinking Water Quality

For the purpose of this study, total coliforms and faecal coliforms were used during the first phase to monitor the general hygiene quality of selected household drinking water of rural and urban areas where both HIV/AIDS and non-HIV/AIDS individuals resided. The water samples were subjected to culture-based methods to ascertain the presence and absence of *Salmonella typhimurium*, *Shigella dysenteriae*, and *Vibrio cholerae*. In the second phase, selected colonies of total and faecal coliforms as well as those of the target pathogens were exposed to biochemical tests, followed by a molecular study for the confirmation of all target pathogenic bacteria including *Escherichia coli*. The third phase of the study established the presence of the rotaviruses in household drinking water of both rural and urban areas. The last phase of this study focused on the risk assessment to determine directly or indirectly the potential impact of the pathogenic *Escherichia coli*, *Salmonella typhimurium*, *Shigella dysenteriae*, and *Vibrio cholerae* and rotaviruses on public health, especially in immunocompromised individuals such as HIV/AIDS patients presenting with diarrhoeal diseases.

#### 2.3.1. Assessment of Sanitary Quality of Drinking Water in the Target Areas

Detection and enumeration of total and faecal coliforms were determined by membrane filtration techniques according to standard methods [34] using coliform Chromocult Agar (Merck, Johannesburg, South Africa). The mFC agar and MacConkey agar (Merck, Johannesburg, South Africa) were also used as additional media for the isolation of faecal coliforms. Following the enumeration, five colonies with same morphological characteristics were randomly selected from the plates for presumptive faecal and total coliforms and transferred onto mFC and chromocult media by the streak-plate method and incubated as indicated above for 24 h. The colonies were further purified by the same method at least three times using nutrient agar (Biolab, Johannesburg, South Africa) before Gram staining. Oxidase tests were then performed on those colonies that were Gram-negative. The API 20E kit was used for the oxidase-negative colonies, and the strips were incubated at 36 ± 1 °C for 24 h. The strips were then analysed, and bacterial species were identified using APILAB Plus bacterial identification software (BioMérieux, Marcy l’Etoile, France). The bacterial counts were calculated and expressed in CFU/100 mL.

#### 2.3.2. Detection of the Presence of Presumptive Pathogenic Bacteria

Presumptive pathogenic *E. coli*, *Salmonella*, *Shigella*, and *Vibrio* spp. were detected according to the standard methods using selective media as described by APHA [34]. Following the detection and enumeration, five characteristic colonies of presumptive *E. coli*, *Salmonella*, *Shigella*, and *Vibrio* spp. were randomly selected from each plate and transferred onto the selective media by the streak-plate method and incubated at 36 ± 1 °C for 24 h. This step was followed by the purification of colonies on nutrient agar plates (Biolab) using the same methods at least three times. The colonies were further subjected to Gram-stain reaction and oxidase production for Gram-negative colonies. The API 20E test kit was used for the oxidase-negative colonies, and the strips were incubated at 36 ± 1 ºC for 24 h. The target presumptive pathogenic bacteria were identified using APILAB Plus bacterial identification software (BioMérieux, Marcy l’Etoile, France). Colonies presumptively identified as target pathogenic bacteria and confirmed to be positive by the API 20E identification system were sub-cultured onto their respective selective media three times before being used for molecular identification.

#### 2.3.3. Molecular Identification of Bacteria *E. coli*, *Salmonella typhimurium*, *Shigella dysenteriae*, and *Vibrio cholerae*

The reference strain for virulent *S. typhimurium* (ATCC 14028) was obtained from MicroBioLogics (Minnesota, USA). For *S. dysenteriae* (ATCC 13313), *V. cholerae* (ATCC 39315), and *E. coli* (ATCC 25922), the reference strains were obtained from the Council for Scientific and Industrial Research (CSIR, Pretoria, South Africa). All these reference strains were confirmed by cultural tests according to the standard methods using their specific selective media [34]. Thereafter, they were maintained in nutrient broth at 36 ± 1 °C for 24 h and preserved in 15% glycerol at −20 °C until use. 

Overnight cultures of each reference bacterial strain and each of the presumptive *E. coli*, *Shigella*, *Salmonella*, and *Vibrio* spp. colonies were prepared in 100 mL nutrient broth, and the incubation was performed at 36 ± 1 °C in a shaking incubator (Scientific Ltd., RSA, Centurion, South Africa). For each target pathogen, 1 mL bacterial cells were concentrated by centrifugation at 10,000 × *g* (8600 rpm·min^−1^) for 5 min using in Heraeus Pico 21 micro-centrifuge (Thermo Scientific, Randburg, South Africa). Thereafter, the pellets were washed twice with sterile molecular-grade water before being suspended in 200 μL sterile Milli-Q water. The DNA extraction was performed using the ZR Fungal/Bacterial DNA Kit (ZYMO Research, Pretoria, South Africa) according to the procedures provided by the manufacturer’s instructions. For each target bacteria, 200 µL of the fresh culture was used to extract the genomic DNA using a ZR//Bacterial DNA Kit™ (Inqaba Biotech, Pretoria, South Africa). 

The amplification of the specific target gene associated with each target pathogenic bacterium was performed using species-specific primers mentioned in Table 1. These primers were synthesised by Inqaba Biotechnical Industries, (Pty) Ltd. (Pretoria, South Africa). The *uidA* gene that encodes for β-D-glucuronidase was used to identify the *E. coli*, while the *IpaB* gene encoding for the invasion plasmid antigen B was used for *S. typhimurium*. As for the detection and identification of *S. dysenteriae* and *V. cholerae*, the *IpaH* gene encoding for the invasion plasmid antigen H and the *EpsM* gene encoding for the component of cytoplasmic membrane protein were used, respectively. The PCR amplification of the target DNA was conducted in a thermal cycler (MJ MiniTM Personal Thermal Cycler, Biorad) using 200 μL PCR tubes and a reaction mixture volume of 50 μL. The reaction mixture consisted of 10 to 20 ng of template DNA, 25 μL 2× Dream TaqTM PCR master mix (10× Dream TaqTM buffer, 2 μM dNTP mix and 1.25 μM Dream TaqTM polymerase), and a 10 μM concentration of each PCR primer. The ultra-pure nuclease-free water was used to make up a total volume of 50 μL for the reaction mixture. 

An aliquot of 10 μL PCR product for each target pathogen was electrophoresed through a 1.5% agarose (*w*/*v*) gel (Merck, RSA) in 1 × TAE buffer (40 mM Tris-HCl, 20mM Na-acetate, 1 mM EDTA; pH 8.5, Biorad) and stained with 0.5 μg·mL-1 ethidium bromide (EtBr, Merck, Midrand, South Africa). The product was visualised under UV light in an InGenius L Gel documentation system (Syngene, Vacutec RSA). A 100-bp ladder (Fermentas, Inqaba, Biotec, South Africa) was added to each gel as a molecular size standard, and the electrophoresis was performed at 80 V for 30 min. For each target bacterial pathogen, a negative control (all PCR reagents except for the template DNA) and positive control (the reference strain of each target pathogen) were also added during each PCR run.

### 2.4. Recovery and Molecular Detection of Rotaviruses

The primer pairs sBeg/End 9 (1062 bp) and Con 2/con 3 (876 bp) were used to reverse transcribe full-length copies of the VP7 gene and VP4, respectively. The G and the P typing was executed using a nested multiplex polymerase chain reaction (PCR) using previously described G-specific and P-specific primers [38]. Briefly, for P genotyping, primers 1T-1D, 2T-1, 3T-1, 4T-1, 5T-1, mP11, and p4943, known to have specificity for the following P types: P(8) P(4), P(6), P(9), P(10), P (11), and P(14), respectively, were added onto the first round of amplification containing primers specific for VP4. Similarly for G genotyping, subsequent to first round of VP7 RT-PCR products, a second-round multiplex PCR containing primer RVG9, as well as primers aBT1, aCT2, mG3, aDT4, aAT8v, mG9, G10, and G12b, specific for G types 1, 2, 3, 4, 8, 9, 10, and 12, respectively.

### 2.5. Quantitative Microbial Risk Assessment (QMRA)

The risk assessment approach has been used as a guide for microbial standards [20]. The microbial risk models used for drinking water were evolved by defining variations in the daily pathogen exposure at the level of the individual consumer drinking water. The β-Poisson model was chosen as it took cognizance of the link between exposure dose and the probability of infection existing in pathogen–host interactions. Moreover, the qualitative data on the isolation of the microorganisms were used to determine the quantitative health assessment. The risks involved with a single and a weekly exposure for different strains of enteric pathogens were calculated. The following equations were used to calculate the daily risk of infection [39].

(1) $$P_{i}=1-{\left[1+\frac{d}{N_{50}}\left(2^{\frac{1}{a}}-1\right)\right]}^{-a}$$

(1) Beta-Poisson Distribution Equation 

And

(2) $$P_{i}=1-e\left(-a^{2}d\right)$$

(2) Exponential Distribution Equation 

where:

*P_i_* = probability (risk) of infection; 

*d* = dose of exposure (number of organisms ingested based on the consumption of 100 mL water per day containing 1 organism, isolated using PCR);

*α* (Alpha) = parameter characterised by the dose–response relationship;

*N*_50_ = median infectious dose. 

For multiple exposures, the following equation was used:

(3) $$P\left(n\right)=1-{\left(1-P_{i}\right)}^{n}$$

where:

*n* is the number of times exposure occurs and

*P_i_* = probability (risk) of infection from a single exposure event.

For example, annual exposure—*n* = 365 and weekly exposure *n* = 52.

## 3. Results

### 3.1. Assessment of Sanitary Quality of Drinking Water in the Study Areas

In this study, faecal and total coliforms were used to assess the sanitary quality of drinking water in the target areas. As can be seen in Table 2, the results revealed the presence of these indicator bacteria in all the drinking water collected from both rural and urban areas. Compared to urban areas, rural areas showed higher concentrations of both faecal and total coliforms, which ranged from <1 to >97 CFU/100 mL and from <10 to >347 CFU/100 mL, respectively. However, in terms of health-based drinking water guidelines, these concentrations of faecal and coliforms in drinking water consumed by the communities from the study areas were above the recommended limits, which are 0 CFU/100 mL

### 3.2. Prevalence of Target Potential Pathogenic Bacteria Based on Culture Methods

Out of 1867 water samples collected from households of the community of Ugu District Municipality, 638 samples (219 from urban and 419 rural) were found to be contaminated. Table 3 illustrates the prevalence of presumptive target bacteria isolated from drinking water provided to rural and urban areas of the Ugu District Municipality. The results revealed that, among the target bacterial species, *E. coli* was found to be the only most frequent and abundant microorganism isolated in drinking water samples collected from various sites in both rural and urban areas of Ugu District Municipality. These bacterial species were found to be at a high prevalence (range: 28.3% to 41%) in drinking water samples collected from rural areas, with Bomela samples having the highest prevalence. In the urban area, *E. coli* was at low prevalence in drinking water samples (ranges: 13.2 to 26.4%). *Salmonella* and *Shigella* were present only in the rural areas, with their prevalence rate dominating in household drinking water samples (from 2.2% to 8.7% and between 4.4% and 9%, respectively). With the culture methods, no *Vibrio* species were found in any of the drinking water samples collected from the rural and the urban areas. Other bacteria such as *Citrobacter*, *Enterobacter*, and *Pseudomonas* species were also frequently found in drinking water samples collected from both rural and urban areas, while *Bacillus* and *Klebsiella* were isolated from the urban (Anneline and Hibberdene) and the rural drinking water (Boboyi, and Bomela) areas, and *Proteus mirabilis* was isolated only in the rural areas. 

### 3.3. Prevalence of the Target Bacterial Species According to Molecular Characteristics and Identification

Out of 638 water samples that were found positive for presumptive bacteria using culture-based methods, 140 isolates (20 per site) were subjected to molecular analysis using species-specific PCR. The results confirmed the presence of pathogenic *E. coli*, *Salmonella typhimurium*, *Shigella dysenteriae*, and *Vibrio cholerae* only in water collected from the rural areas (Table 4). 

### 3.4. Detection and Characterisation of Rotavirus from the Treated Drinking Water

The rotavirus RNA was detected using the single step RT-PCR of the VP7 gene. As can be seen in Table 5, out of 164 water samples subjected to this test, this enteropathogenic virus was only found in two water samples (1.2%), which were collected from the urban areas of Anneline and Port Shepstone. Moreover, this virus was detected in samples collected at the beginning of the study during the dry season. 

### 3.5. The Probability of Infection with S. typhimurium, S. dysenteriae, V. cholerae, E. coli, and Rotavirus

**Figure 1:**
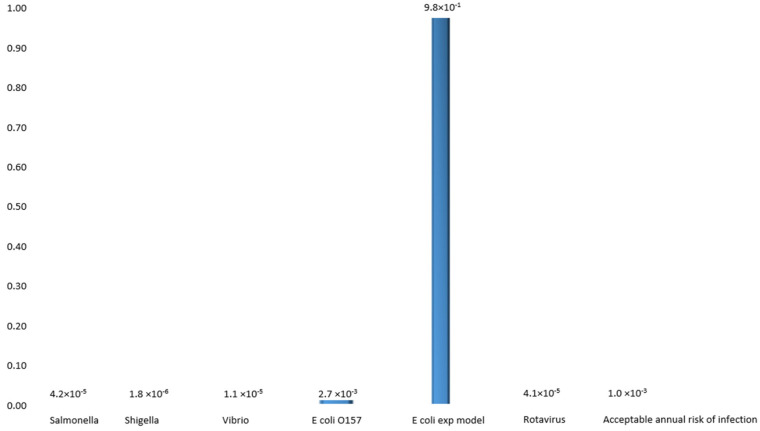
Probability of infection associated with consumption of water infected with a single enteric pathogenic microorganism collected in alternating months.

The bacterial agents responsible to cause intestinal diseases have been linked to but not limited to *S. typhimurium*, *S. dysenteriae*, and *V. cholerae*. For most microorganisms to cause an infection, a dose of 10^6^ to 10^8^ is needed, although this varies according to microorganisms. For example, a few cells of *S. typhi* and *S. dysenteriae* are needed to cause infection, whereas large numbers of cells in the case of *Vibrio* are needed, as indicated in Figure 1 below.

**Figure 2:**
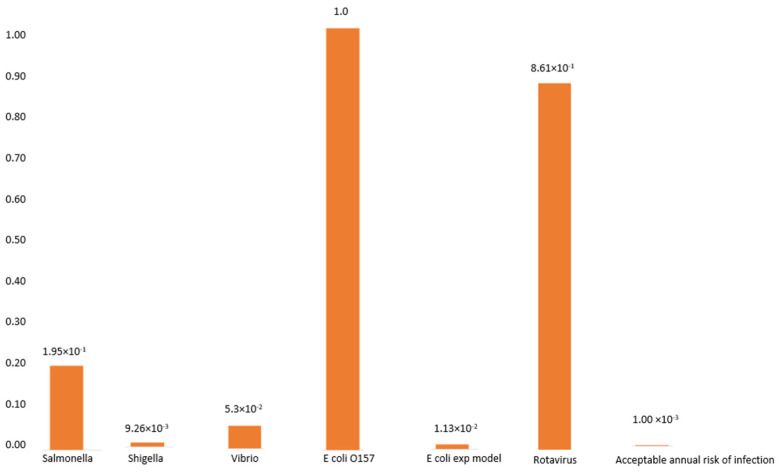
Probability of infection associated with the weekly consumption of water infected with enteric pathogenic microorganisms in the alternating months of November–August.

In the case of the Ugu population, household water samples were found to contain bacterial pathogens, and study participants were assumed to be exposed to a single organism on the basis of the detection of each of the pathogenic organisms detected in the water. The probability of infection for *E. coli* O157 (2.7 × 10^−3^ or 9.8 × 10^−1^), *Salmonella typhi* (4.2 × 10^−5^), *Shigella* spp. (1.8 × 10^−6^), *Vibrio* spp. (10 × 10^−5^) and rotavirus (4.1 × 10^−1^) resulting from a single event are illustrated in Figure 2, whereas if weekly consumption is assumed, the probability of infection would increase to 1.0 × 10^0^, 1.95 × 10^−1^, 9.26 × 10^−3^, 5.02 × 10^−2^, and 8.61 × 10^−1^, respectively (Figure 2).

### 3.6. The Probability of Infection with Diarrheagenic E. coli

Studies on the infection with *E. coli* have been done elsewhere [40], wherein the risk of infection from 1mL of water was found to range between 0 and 74% [40]. To have 19% probability of infection, an individual would need to just ingest 1 diarrhoeagenic *E. coli*, and if this is repeated every week, the probability of infection would increase to 100%, as illustrated in Figure 2.

#### The Probability of Infection with Rotavirus in Water

Drinking water contaminated with rotavirus resulted in a probability of infection of a single event of exposure to one virus of 0.037 (close to 4%). There was no rotavirus isolated from household water collected from rural areas. A similar genotype (G1) was isolated in both urban areas with different serotypes of P (8) and P (6) from Anneline and Port Shepstone, respectively.

## 4. Discussion

Contaminated water resources have a detrimental effect on the environment and human health [41]. Previous studies have found that factors such as a number of organisms consumed, age, sex, nutrition, and CD4 count affect the risk of infection in an individual [1,41]. Furthermore, polluted water not only causes human suffering, but it also affects the economy. The assessment of the cost of water-related enteric illness has been reported in developing regions and, on average, an estimated that 100 people are losing their lives per annum, resulting in large economic costs associated with it [8]. In South Africa, the control of water quality still remains a challenge, especially in terms of financial stability of service providers, thus leading to less attention paid to maintenance. In this study, the prevalence and health risks associated with the consumption of contaminated water in rural areas of the Ugu District Municipality were assessed. As current drinking water standards emphasise the detection of microbial pollutants, it became important to investigate the risk presented by microbes found in drinking water polluted with human and animal excreta, which may directly or indirectly affect the health of those who consumed this water source [42].

In the first stage, the study focused on total coliforms and *E. coli*, which are considered not only as the indicator bacteria, but are also referred to as target bacteria, while other bacteria that may be present in drinking water are called non-target bacteria. The indicator bacteria may have health effect that can range from no physical impact to several illness such as gastrointestinal illness. In rare cases, contaminated water may also result in significant illness harmful effects. Although total coliforms alone are not likely to cause illness, in contrast, their presences in drinking water are known as an indication of water supply contamination by harmful microorganisms. In this study, a number of other bacteria were also frequently found in drinking water used by both rural and urban areas for multiple purposes (Table 2). The presence of the non-target microorganisms (*Pseudomonas*, *Enterobacter*, *Citrobacter*) may potentially become opportunistic microorganisms capable of causing harm to individuals with reduced immunity.

Results also revealed the occurrence of enteropathogenic microorganisms above the acceptable limit (0 CFU/100 mL) for domestic use. As can be seen in Table 2, there is a considerable variation in the pathogen exposure through the water to cause illness. People living in the target rural areas have a high risk of being exposed to infections if drinking water of good quality is not provided to these communities. The infected individuals are at an even higher risk as exposure to poor quality water will exacerbate their illness. Results, therefore, revealed that culture-based techniques give an indication of the general microbial profile, whilst the molecular analyses are sensitive and specific and provide conclusive evidence of the microorganisms under investigation.

In the present study, genomic DNA of randomly selected water isolates was subjected to conventional PCR using species-specific primers to confirm the presence of the target organisms in the drinking water. The outcome of the PCR-based analysis revealed the absence of *Salmonella typhimurium* and *Shigella dysenteriae* in the urban areas and 65% in the samples collected from the rural areas (Boboyi, Bomela, and Gamalakhe). *Salmonella*, *Shigella*, *Vibrio*, and *E. coli* 0157 were enteric pathogens identified in final treated water collected from urban and rural houses. The presumptive *Vibrio* isolated conventionally from the urban area was not confirmed with the molecular analysis, whilst the ones from the rural areas was confirmed.

Methods to determine the risk of infection associated with the microbiological quality of the water have been developed. These include the volume of water ingested and the concentrations of pathogenic microorganisms in water [1]. In the present study, using the Beta Poisson analysis, it was found that different microorganisms require different concentrations to cause infection. It is possible that 100 mL of contaminated water can contain only a few microorganism cells capable of causing infection. This is translated to the fact that one organism is needed to trigger the infection from as little as 1 mL of water ingested. The probability of infection with the identified pathogens was taken into consideration, and it was found that the probability of infection according to a single ingestion rate was as followed: (2.7 × 10^−3^ or 9.8 × 10^−1^), (4.2 × 10^−5^), (1.8 × 10^−6^), (10 × 10^−5^), and (4.1 × 10^−1^) for *E. coli*, *Salmonella typhimurium*, *Shigella* sp., *Vibrio* sp., and rotavirus, respectively.

The presence of pathogens such as *V. cholerae*, *S. typhimurium*, and *S. dysenteriae* in water can pose serious health risks if a concentration above the lowest infection-causing dosage (*Salmonella* = between 10^3^ and 10^5^, *Shigella* = between 10 and 100, and *Vibrio* = 10^3^) is ingested, whilst the presence of *E. coli* is known as an indication of contamination with human and animal excretions. People with weaker immune systems will be placed at a much higher risk of infection than individuals with strong immunity. The present study has shown the risks associated with drinking water contaminated at the household level. Even though other studies have shown that disinfecting water would make it safe for human consumption, it was also shown that it is not always guaranteed that treated water guarantees pathogen-free drinking water [1]. In rural areas of developing countries where the quality of drinking water is not really guaranteed, there is a need to use other methods such as boiling water that is known to eliminating pathogens.

It is reported that *Salmonella*, *Shigella*, *E. coli* [43], and viruses [44] have been isolated in immunocompetent and in immunocompromised individuals. Assuming that immunocompromised and immunocompetent individuals had consumed the same amount of water, the infection rate will be higher in immunocompromised individuals compared to the latter. This has been previously confirmed in a study undertaken by CDS [44], whereby a higher rate of infection was observed in individuals with HIV/AIDS after consumption of water contaminated with enteric pathogens. Diarrhoea in immunocompromised individuals presents a challenge as classic and opportunistic microorganisms become potentially pathogenic as a result of defective immunity. Therefore, if the diarrhoea is not controlled, it becomes the cause of morbidity and mortality in immunocompromised individuals and children aged less than five. In most cases, *Shigella* species has been found to be the most causative agent of diarrheal in immunocompromised individuals globally. Even though the importance of infection in various serotypes is not understood, it was reported that 30% of these infections were caused by *S. dysenteriae* [45]. The overall mortality rate associated with infections in developed countries was less than 1% [45]. In the Far East and the Middle East, mortality rates for *S. dysenteriae* infections were found to be as high as 20 to 25% in the regions, with the most common serotype in South Africa being *S. flexneri*, which was predominantly isolated in invasive cases in hospitals [46]. It was found that *Salmonella* is the commonly isolated microorganism in HIV/AIDS individuals compared to immunocompetent individuals, and the death rates were higher in this group than in immunocompetent individuals [47]. The high death rates were due to toxins excreted by the bacteria (cytotoxin and enterotoxin), which damage the intestinal cells, rendering the body unable to retain and absorb fluids, resulting in diarrhoea. In individuals with a competent immune system, the stomach acid kills the bacteria on contact. The same mechanism is applicable to *Pseudomonas* infection; in normal immune individuals, the organism is killed by acids in the stomach. However, in the case of immunocompromised individuals, severe infections are established at the sites of epithelial tissue lining cavities of the digestive system. One outstanding characteristic of *P. aeruginosa* infection in the HIV-infected community is the prevalence of community-acquired versus nosocomial infection. It is documented that viruses that cause diarrhoea can be isolated in contaminated water and stools of infected individuals [47,48].

Viral infections are usually self-limiting in healthy individuals but can cause high morbidity in children under the age of five, the elderly, immunocompromised people, and pregnant women [48]. Rotavirus was isolated in the present study in water samples. Molecular approaches using the single-step RT-PCR for the detection and characterisation of rotaviruses in the treated water supplied by the Ugu District municipality to rural and urban areas and from the diarrhoeic stools of patients attending the Port Shepstone and Murchisson hospitals were applied. The results revealed the common G type in the finished treated water collected from the urban areas, and none from the water collected from rural areas. The G types isolated fully correlate with strains isolated previously [49,50,51,52,53]. A different type, G1(P4), was isolated in KwaZulu-Natal. Using the Poisson distribution, we determined that individuals were at a higher risk of being infected with rotavirus than other pathogens, with a probability of infection of 0.037 being significantly greater than the acceptable annual risk of 0.0001. The present study revealed that people are at risk of developing an infection as the number of cells present in the water exceeded the annual acceptable limit calculated using the Poisson method.

## 5. Conclusions

This study has shown that contaminated water could pose significant health risks to the Ugu District Municipality community. The estimated risks for the different strains of enteric pathogens are higher than the acceptable annual risk, with rotavirus causing the greatest risk of infection. It is well known that immunocompromised individuals have a varied vulnerability to infections, and therefore are at higher risk of infections. Thus, a continuous assessment of the status of drinking water in such areas needs to be performed to determine which bacteria and viruses are circulating in the community and their potential to cause an epidemic. The study calls for the government to intensify the programs that will educate the community on proper water storage and hygiene as well as to use other drinking water treatment such as boiling to avoid infection and cross-contamination.

## Figures and Tables

**Table 1:** PCR oligonucleotide primers used to detect *E. coli*, *Salmonella*, *Shigella*, and *Vibrio*.

Pathogens	Gene Targeted	Sequences	Amplicon Size	Ref.
*E. coli*	*uidA*	F-AAAACGGCAAGAAAAAGCAG- R-ACGCGTGGTTAACAGTCTTGCG-	147	[35]
PCR Steps	Temperature (°C)	Duration	Number of cycles	
Initial denaturation	94	2 min	35	
Denaturation	94	1 min		
Annealing	58	1 min		
Extension	72	1 min		
Final extension	72	5 min		
*Salmonella*	*IpaB*	F-GGACTTTTTAAAAGCGGCGG- R-GCCTCTCCCAGAGCCGTCTGG	314	[36]
PCR Steps	Temperature (°C)	Duration	Number of cycles	
Initial denaturation	94	2 min	25	
Denaturation	94	1 min		
Annealing	62	1 min		
Extension	72	2 min and 5 s		
Final extension	72	7 min		
*Shigella*	*IpaH-U1* *IpaH-L1*	-CCTTTTCCGCGTTCCTTGA- -CGGAATCCGGAGGTATTG C-	199	[37]
PCR Steps	Temperature (°C)	Duration	Number of cycles	
Initial denaturation	95	5 min	40	
Denaturation	95	30 s		
Annealing	60	1 min		
Extension	72	1 min		
Final extension	72	7 min		
*Vibrio*	EpsM	F-GAATTATTGGCTCCTGTGCAGG- R-ATCGCTTGGCGCATCACTGCCC-	248	[36]
PCR Steps	Temperature (°C)	Duration	Number of cycles	
Initial denaturation	94	2 min	35	
Denaturation	94	1 min		
Annealing	58	1 min		
Extension	72	2 min and 5 s		
Final extension	72	4 min		

**Table 2:** Overall microorganism counts detected in drinking water samples in the study area.

Study Area		Faecal Coliforms CFU/100 mL	Total Coliforms CFU/100 mL
Rural	Boboyi	Min < 2 Max > 97	Min < 11 Max > 347
	Bomela	Min < 2 Max > 88	Min < 17 Max < 220
	Gamalakhe	Min < 0 Max > 64	Min < 10 Max < 219
Urban	Anneline	Min < 1 Max > 12	Min < 5 Max < 104
	Hibberdene	Min < 1 Max > 66	Min < 1 Max > 161
	Margate	Min < 0 Max > 2	Min < 1 Max > 214
	Portshepstone	Min < 0 Max > 3	Min < 0 Max >199

**Table 3:** The prevalence of selected bacterial species in drinking water of the study areas in the Ugu District Municipality, KZN.

Species	Study Sites						
	Boboyi	Bomela	Gamalakhe	Margate	Hibberdene	Anneline	P/Shepstone
*Bacillus* spp.	7 (7.7%)	3 (3.3%)	0(0%)	0(0%)	0(0%)	1 (1.02%)	0 (0%)
*Citrobacter* spp.	9 (9.9%)	12 (13.2%)	20 (22.0%)	16 (17.6%)	12 (13.2%)	24 (26.4%)	24 (26.4%)
*Enterobacter* spp.	12 (13.2%)	2 (2.2%)	16 (17.6%)	12 (13.2%)	32 (35.2%)	16 (17.6%)	8 (8.8%)
*E. coli*	25 (27.5%)	38 (41.8%)	34 (37.4%)	16 (17.6%)	18 (19.8%)	12 (13.2%)	24 (26.4%)
*Klebsiella* spp.	4 (4.3%)	12 (13.2%)	0 (0%)	0 (0%)	2 (2.2%)	0 (0%)	0 (0%)
*Salmonella* spp.	8 (8.8%)	2 (2.2%)	6 (6.6%)	0 (0%)	0 (0%)	0 (0%)	0 (0%)
*Shigella boydii*	12 (13.2%)	9 (9.9%)	4 (4.4%)	0 (0%)	0 (0%)	0 (0%)	0 (0%)
*Pseudomonas* spp.	10 (11.0%)	13 (14.3%)	11 (12.1%)	8 (8.8%)	12 (13.2%)	12 (13.2%)	16 (17.6%)
*Proteus mirabilis*	4 (4.4%)	0 (0%)	0 (0%)	0 (0%)	0 (0%)	0 (0%)	0 (0%)
Total *n* = 638	91/91 (100%)	91/91 (100%)	91/91 (100%)	52/91 (57%)	76/91 (84%)	65/91 (71%)	72/91 (79%)

**Table 4:** Detection of target bacterial pathogens in drinking water samples by species-specific PCR.

Bacterium	Sampling Points						
Rural (*n* = 20 Per Site)				Urban (*n* = 20 Per Site)			
	Boboyi	Bomela	Gamalakhe	Anneline	Hibberdene	Margate	P/Shepston
*E. coli*	12 (60%)	9 (45%)	6 (30%)	0 (0%)	0 (0%)	0 (0%)	0 (0%)
*Salmonella typhimurium*	13 (65%)	8 (40%)	8 (40%)	0 (0%)	0 (0%)	0 (0%)	0 (0%)
*Shigella dysenteriae*	6 (30%)	8 (40%)	0 (0%)	0 (0%)	0 (0%)	0 (0%)	0 (0%)
*Vibrio cholerae*	8 (40%)	8 (40%)	0 (0%)	0 (0%)	0 (0%)	0 (0%)	0 (0%)

**Table 5:** Detection and characterisation of rotavirus from the treated drinking water collected from the urban areas (water samples *n* = 164).

Source	Type of Sample	Genotype
Tap water (Anneline)	Final treated water sample	G1P (8)
Tap water (P/Shepstone)	Final treated water sample	G1P (6)
